# Are PCI Service Volumes Associated with 30-Day Mortality? A Population-Based Study from Taiwan

**DOI:** 10.3390/ijerph14111362

**Published:** 2017-11-09

**Authors:** Tsung-Hsien Yu, Ying-Yi Chou, Chung-Jen Wei, Yu-Chi Tung

**Affiliations:** 1 Department of Health Care Management, National Taipei University of Nursing and Health Sciences, Taipei 108, Taiwan; ericthyu@ntu.edu.tw; 2 Institute of Health Policy and Management, National Taiwan University, Taipei 100, Taiwan; s202280002@gmail.com; 3 Department of Public Health, Fu-Jen Catholic University, Taipei 242, Taiwan; 034425@mail.fju.edu.tw

**Keywords:** service volume, PCI, multilevel analysis, comparison

## Abstract

The volume-outcome relationship has been discussed for over 30 years; however, the findings are inconsistent. This might be due to the heterogeneity of service volume definitions and categorization methods. This study takes percutaneous coronary intervention (PCI) as an example to examine whether the service volume was associated with PCI 30-day mortality, given different service volume definitions and categorization methods. A population-based, cross-sectional multilevel study was conducted. Two definitions of physician and hospital volume were used: (1) the cumulative PCI volume in a previous year before each PCI; (2) the cumulative PCI volume within the study period. The volume was further treated in three ways: (1) a categorical variable based on the American Heart Association’s recommendation; (2) a semi-data-driven categorical variable based on k-means clustering algorithm; and (3) a data-driven categorical variable based on the Generalized Additive Model. The results showed that, after adjusting the patient-, physician-, and hospital-level covariates, physician volume was associated inversely with PCI 30-day mortality, but hospital volume was not, no matter which definitions and categorization methods of service volume were applied. Physician volume is negatively associated with PCI 30-day mortality, but the results might vary because of definition and categorization method.

## 1. Introduction

People always look for information about their health and healthcare and about whether healthcare services are needed by making deliberate healthcare choices. However, healthcare is unique and distant compared to other industries; the asymmetry of information between provider and patient is well documented.

Compared to other information, service volume is a much easier indicator for understanding the quality of the healthcare provider. However, can people rely only on this indicator to make healthcare choices? In 1979, Luft and his colleagues published a classic article to explore the relationship between service volume and outcomes; they found that higher service volume might lead to better outcomes [1]. The results prompted research interest in the volume-outcome relationship and triggered further research on several related topics.

In the literature, percutaneous coronary intervention (PCI) has been one of the most important procedures in recent decades. Some studies have discussed whether high hospital PCI volume was associated with a lower mortality rate, but the findings were inconsistent [2,3]. Regarding physician volume, the findings were controversial as well [4]. In addition, several groups such as the American Heart Association [5] and Leapfrog [6] also proposed a minimum volume for different purposes.

The controversial findings can perhaps be attributed to four main factors. First of all, the categorization methods were not consistent [7]. In the past, researchers categorized provider volume using different and subjective methods such as quartile [2,8], quintile [4], and the expert consensus, i.e., American Heart Association’s recommendations [3,4,9]. The heterogeneity of categorization methods might have produced different results [10,11,12]. Secondly, the definitions of service volume have varied [10,11]. Most studies usually calculated the cumulative service volume within the study period/current-year volume. A potential argument for using the current-year volume is that the current-year volume and prior-year volume are highly correlated. However, the provider (hospital and surgeon/physician) volume may change over time [13]. Furthermore, the current-year volume may not reflect the provider’s level of experience at the time a patient received healthcare services. It also violates the assumption of ‘practice makes perfect’. Finally, the statistical methods used in earlier studies might be inappropriate. Most studies ignored the issue of clustered or nested data. The clustering or nesting phenomenon implies that subjects in a group are from the same population such as students in a class or classes in a school. Such clustering suggests a certain amount of homogeneity among the subjects, thus violating the assumption of independent samples in conventional regression analysis. Ignoring this issue may lead to incorrect conclusions. Recent studies have already taken this into account and adopted appropriate statistical methods such as generalized estimating equations (GEEs) [14], random effects, or hierarchical linear modeling [2] to deal with this issue. Plus, appropriate risk adjustment is also an important issue to take into account, especially in physician- and hospital-level studies [10,15].

In Taiwan, Lin et al. studied this topic as well [9], and they focused on the relationship between hospital volume and mortality. The American Heart Association’s recommendation was adopted in their work to categorize hospital volume; they found that patients who were treated at a low volume hospital had a higher mortality risk. The effect of physician volume was unclear. Furthermore, a reduction in the variability of PCI volumes in Taiwan comes with the medical technology developed. Therefore, it is necessary to re-examine the relationship between PCI volumes and outcome. In view of the heterogeneity of service volume definitions and categorization methods, no existing studies have used them simultaneously to explore the relationship between volume and outcome. Further, only a few studies compared the effects of hospital and physician volume with the simultaneous outcome of PCI care. Therefore, this study used two definitions of volume and adopted three types of categorization method to examine whether the relationship between PCI volume and outcome existed.

## 2. Methods

### 2.1. Ethics

The protocol for this study was approved by the Institutional Review Board of the National Taiwan University Hospital (protocol #201412074W). The dataset we used in this study was secondary data; all information was de-identified by data owners.

### 2.2. Study Design

This retrospective and cross-sectional study adopted a multilevel design to examine the relationship between provider volume and 30-day mortality after adjusting for patient-, physician-, and hospital-level covariates.

### 2.3. Data Source

We used data from the Taiwan National Health Insurance Research Database (NHIRD). The NHIRD, published by the Taiwan National Health Research Institute, includes all the original claims data and registration files for beneficiaries enrolled under the National Health Insurance (NHI) program. The database covers the 23 million Taiwanese enrollees (approximately 98% of the population) in the NHI program. It is a de-identified secondary database containing patient-level demographics and administrative information. The data are released for research purposes.

### 2.4. Study Population and Exclusion Criteria

We restricted our analysis to hospitalizations in which a patient had a procedure code that indicated a percutaneous coronary intervention (International Classification of Diseases, ninth edition, Clinical Modification (ICD-9-CM), codes 36.00 to 36.06 and 36.09) [9] in 2009. We excluded patients with missing data for gender (*n* = 22), and under 18 years (*n* = 2) of age to restrict our evaluation to an adult population.

### 2.5. Dependent Variable

The dependent variable in this study was the 30-day mortality from any cause after hospitalization for PCI; 30-day mortality was determined by linking inpatient admission records with the withdrawal certificate records. The only reason for being withdrawn from NHI coverage within 30 days of hospital admission would be death. Withdrawal dates are the same as the date deceased according to the death certificate [16,17].

### 2.6. Independent Variable

In this study, we used the following two definitions to define service volumes: (1) cumulative service volumes by each physician and hospital in the 12 months before the index procedure for each treatment [18] and (2) the cumulative operation volumes by each physician and hospital within the study period, which was the most common definition in the literature. For the former, we calculated the monthly service volume for each physician and hospital first, summed up the previous 12 monthly service volume before the index procedure, and then calculate the average annual volume for all physicians/hospitals. Theoretically, the former definition can better reflect the provider’s level of experience at the time a patient receives healthcare services.

The current work treated operation volumes in three different ways: (1) a binary variable based on the American Heart Association’s recommendation that a hospital’s annual volume ≥200 and a physician’s ≥50 should be used as cut-off points [5]; (2) a predetermined data-driven categorical variable based on k-means clustering algorithm. This study categorized physician and hospital volume into low, medium, and high volume groups by a quartile method and k-means clustering algorithm; and (3) a non-predetermined data-driven categorical variable based on a generalized additive model.

K-means clustering is an unsupervised machine-learning algorithm introduced by MacQueen in the 1960s. This method is not only a simple and very reliable method in categorization/classification, but it is also recognized as one of the top 10 algorithms in data mining [19]. The main idea of this method is to partition observed data points into k non-overlapping clusters by minimizing the within-group sum of squares. Each point is assigned to the mean of its cluster using the Euclidian distance. Firstly, k cluster centers were randomly generated. Previous studies usually divided physicians and hospitals into low-, medium-, and high-volume groups; therefore, we also predetermined the physician and hospital service volume into three groups (k = 3). Then the participants were assigned to the cluster with the shortest distance to these cluster centers. Finally, the cluster centers were recomputed using the new cluster assignment, and these steps were iterated until convergence was achieved.

The Generalized Additive Model (GAM) was proposed by Hastie and Tibshirani in 1990 [20]. The main feature of GAM is that it does not require many assumptions (i.e., normal assumption, variance homogeneity), and it is more capable of dealing with non-linear data. As long as the distribution of the dependent variable is within an exponential family such as a normal, binomial, Poisson, or gamma distribution, a GAM can be applied [21]. In this study, the GAM method divided the service volume into three groups.

However, our data was skewed and did not follow a normal distribution. Therefore, we conducted logarithmic transformations on the service volumes, except the American Heart Association’s recommendation. The cut-off points of each method using two different definitions to define service volume were presented in Table 1.

### 2.7. Covariates

In addition to physician and hospital volumes and 30-day mortality, we collected patient-, physician-, and hospital-level data. Firstly, the patient-level variables included age, gender, income status, Deyo’s Charlson comorbidity index, number of vessels obstructed, stent use, history of coronary artery bypass graft (CABG) surgery, and PCI treatment [4]. Secondly, the physician-level variables included age and gender. Thirdly, the hospital-level variables included hospital ownership, accreditation status, and geographic location.

### 2.8. Statistical Analysis

All statistical analyses of the volume-outcome relationship, k-means clustering, and Generalized Additive Model were performed using SAS 9.4 (SAS Institution Inc., Cary, NC, USA). In statistical testing, a two-sided *p* value ≤ 0.05 was considered statistically significant. The distributional properties of the continuous variables were expressed by mean ± standard deviation (SD), whereas the categorical variables were represented by frequency and percentage. In the univariate analysis, the potential three-level predictors of 30-day mortality were examined using a chi-square test or two-sample *t*-tests, as appropriate. Next, to account for the correlations of physician (level-2) and hospital (level-3), multivariate analysis was conducted by fitting random intercept logistic regression models to each patient’s data to estimate the effects of three-level predictors on the probability of 30-day mortality. Last, a stepwise variable selection procedure was performed to avoid multicollinearity.

## 3. Results

A total of 34,193 cases were included in this study, which received treatment by 1318 physicians in 79 hospitals. The descriptive analysis (see Table 2) showed that the majority of patients were male (73.42%), and the mean age of the patients was 65.73 years. Most patients had one vessel obstructed (72.15%), around 30% and 0.5% of patients had received PCI and CABG, respectively, and around 66% of patients received a stent.

Most patients received PCI treatment by male physicians, with a mean age of 43.57 years, and the average physician service volume for each PCI treatment within the study period was 120, while the average cumulative service volume in the 12 months before each PCI treatment was 116. The data also showed that almost all patients received PCI treatment in medical centers and regional hospitals, and, regarding hospital ownership, in public and non-for-profit hospitals. The average hospital service volume for each PCI treatment within the study period was 814, while the average cumulative service volume in the 12 months before each PCI treatment was 776.

Table 3 shows the distribution of hospital and physician volume. With the American Heart Association’s recommendation, the results showed that around 90% of patients were treated by high volume hospitals, and around 75% of patients were treated by high volume physicians, no matter which definition was used. Regarding the k-means algorithm, although the results also revealed that most patients were treated in high volume hospitals and by high volume physicians, the percentage varied with service volume definition. When the first definition was applied, 94.9% of patients were treated in high volume hospitals, 5% were treated in medium volume hospitals, and only 0.1% were treated in low volume hospitals. As for physician service volume, 74.8% were treated by high volume physicians, 22.2% by medium volume physicians, and 0.3% by low volume physicians. However, when the second definition was applied, 75.6%, 23.9%, and 0.5% of patients were treated in high-, medium-, and low- volume hospitals, respectively, and 81.9%, 15.4%, and 2.7% of patients were treated by high-, medium-, and low- volume physicians, respectively. With the GAM method, the results were different from those with the other methods. When the first definition was applied, the results showed that most patients were treated in medium volume hospitals (57.3%) and by high volume physicians (84.7%). When the second definition was applied, the data also showed that around 90% of patients were treated by high volume physicians, but around 60% of the patients were treated in medium volume hospitals. Since the study population was distributed extremely unequally, therefore, we merged the low and medium volume categories to reduce the effect of imbalance in the multivariate analysis.

Table 4 demonstrates the results of the random intercept model, but only the hospital volume and covariates were placed into the model. However, the results show that the hospital volume was not associated with 30-day mortality, after adjusting for the patient’s age, comorbidity status, stent use, prior PCI history, physician’s age, hospital ownership, and geographic location, no matter which definition was used.

Table 5 demonstrates the results of the random intercept model, while physician and hospital volume were put into the model. When service volume was defined as the cumulative volume in the previous year (Definition 1), the results of Model 1 (AHA’s recommendation) showed that patients who received PCI from low volume physicians had a morality risk 2.34 (95% C.I.: 1.92 to 2.84) times higher than those that received PCI from high volume physicians. However, the results also revealed that hospital volume was not associated with mortality. In Model 2 (k-means), the results also showed that patients who received PCI from low volume physicians had a morality risk 2.37 (95% C.I.: 1.95 to 2.87) times higher than those that received PCI from high volume physicians. An association between hospital volume and mortality was not found here either. In Model 3 (GAM), the results also revealed that patients who received PCI from low volume physicians had a morality risk 2.75 (95% C.I.: 2.27 to 3.31) times higher than those that received PCI from high volume physicians. An association between hospital volume and mortality was still not found.

When service volume was defined as the cumulative volume within the study period (Definition 2), the results also demonstrated that only physician volume was negatively associated with 30-day mortality, but hospital volume was not. Patients who received PCI from low volume physicians had greater a mortality risk than those that received PCI from high volume physicians. The odds ratios were 2.73 (95% C.I.: 2.22 to 3.34) for model 1, 2.78 (95% C.I.: 2.29 to 3.39) for model 2, and 3.15 (95% C.I.: 2.59 to 3.83) for model 3. Finally, in terms of model fitting, we found that Model 3 (GAM) had the best model fitting, no matter which definition was applied.

To understand the association between physician volume and hospital volume, stratified analyses were conducted. Table 6 shows that, when the AHA recommendations were used for hospital classification, patients who received PCI from low volume physicians had a greater mortality risk (OR: 2.15 to 2.77). When the k-means algorithm was applied (Table 7), the results also demonstrated that patients who received PCI from low volume physicians had a greater mortality risk (OR: 2.40 to 2.85), but the results also showed that the physician volume was not associated with 30-day mortality in low volume hospitals (OR: 1.65, 95% C.I.: 0.82 to 3.34). Finally, when GAM was applied (Table 8), the results were the same as above (OR: 2.60 to 4.93).

## 4. Discussion

This nationwide, population-based study was the first, to our knowledge, to use different definitions and categorization methods to explore the volume-mortality relationship in percutaneous coronary intervention, and we also examined whether the hospital or physician volume matters. There were three major findings in this study. First of all, the results of our study demonstrated that hospital volume was not associated with PCI mortality, regardless of the definitions and categorization methods of service volume. However, in terms of physician volume, the results showed that service volume was negatively associated with mortality, no matter which definitions and categorization methods of service volume were applied. The results also implied that physician volume might be more important than hospital volume. Previous findings were controversial; for example, Hannan et al. [22], Vakili et al. [23], and Badheka et al. [2] found that hospital and physician volume were partially negatively associated with mortality. However, another study by Hannan et al. [24], published in 2005, found that the association did not exist. The major difference in these studies was the categorization method because they divided hospital and physician volume into three to five groups based on different cut-off points. In our studies, all methods categorized hospital and physician volume into two groups; therefore, we can compare the influences of different cut-off points with different definitions. No matter which cut-off points were used with different definitions, the results implied that an experienced physician is the key to ensure the outcome of the care, rather than the hospital.

Secondly, which threshold was recommended in this study? Three approaches were adopted in this study for threshold selection. The AHA recommendation was established by expert opinion, k-means only considered the phenomenon of data clustering, and the association between volume and outcome was used for identifying the threshold in GAM. In terms of model fitting, when GAM was applied, the model fitting was better. Therefore, GAM was recommended for threshold selection. Previous studies usually used more arbitrary methods to identify volume thresholds, but the methods we used in this study might provide a solution to optimize local healthcare systems.

Thirdly, the phenomenon of ‘practice makes perfect’ exists in physician behavior. Although several existing studies suggested that high volume physicians could have better outcomes for PCI care, most of these studies adopted the cumulative volume with the study period. This approach is easy to adopt, but it does not reflect the experience (service volume) of a hospital or a physician at the time when a patient receives healthcare services [18]. Theoretically, the cumulative volume before each procedure can better reflect the provider’s level of experience at the time a patient receives healthcare services. However, the timeframe of our data is not long enough to calculate overall career service volume for each physician and hospital, and one-year service volume might not be sufficient to represent the experience of a physician and hospital. Such a limitation is inevitable.

Fourthly, should we centralize patients to a high volume hospital or physician? In addition to ‘practice makes perfect’, ‘selective referral’ is another hypothesis to explain the volume-outcome relationship [25,26]. Several agencies make recommendations for minimum service volume [6,27], and many studies have discussed whether patients should be centralized to high volume hospitals or physicians [28,29,30]. Although this study found that patients who were treated by higher volume physicians had better outcomes, the capacity of hospitals and the workload of physicians should be the primary concern before health authorities turn research findings into real-world policy. In other words, the government and stakeholders should not only set the minimum volume for hospitals and physicians, but should also set the maximum volume. Existing studies have proved that fatigue and overwork might cause severe patient safety events and managerial issues [31,32]. Although this issue is out of the scope of this study, its influence should still not be ignored by policy makers, and it is an important issue for future research.

In our study, a multilevel analysis was applied to manage the nesting issue in the data, and four different methods were adopted to examine the relationship between PCI service volume and 30-day mortality, but the study still suffered from two limitations. Firstly, the NHIRD lacks clinical information such as vessel characteristics, health status, type of PCI (elective or urgent), etc. Therefore, we may have lost some important aspects of the patient profiles that would have influenced the results. Secondly, this study cannot test all cut-off points in the existing literature. While the selection of cut-off points in previous studies varied, it is difficult for this study to test the influences of all cut-off points; therefore, they were represented by the four types of categorization methods that we selected. However, because the findings in this study are consistent, this might not be a limitation.

## 5. Conclusions

In conclusion, we found that physician volume is associated inversely with PCI 30-day mortality, no matter which definitions and categorization methods of service volume were applied. However, an association between hospital volume and PCI 30-day mortality was not found in this study.

## Figures and Tables

**Table 1:** Cut-off points among categorization methods with different definitions of service volume.

	American Heart Association (AHA)	K-Means	Generalized Additive Model (GAM)
**Definition 1**			
Hospital	200	1.8 (6), 4.9 (131)	5.3 (193), 7.0 (1051)
Physician	50	1.1 (3), 3.9 (50)	0.0 (1), 3.4 (30)
**Definition 2**			
Hospital	200	4.2 (68), 5.9 (365)	5.3 (210), 7.0 (1126)
Physician	50	1.1 (3), 3.6 (37)	0 (1), 3.2 (24)

Definition 1: the cumulative service volumes by each physician and hospital in a previous year for each treatment. Definition 2: the cumulative operation volumes by each physician and hospital within the study period. Volumes were transformed into logarithmic values except the American Heart Association’s recommendation.

**Table 2:** Descriptive statistics (*n* = 34,193).

Variables	
Patient Characteristic	
Male, n (%)	25,103 (73.42)
Age, mean (S.D)	65.73 (12.24)
Elixhauser Comorbidity Index, mean (S.D)	1.25 (1.49)
Low income (yes), n (%)	348 (1.02)
Stent, n (%)	
No use	11,289 (33.02)
Bare metal stents	14,113 (41.27)
Drug eluting stents	8791 (25.71)
Vessel obstructed, n (%)	
One vessel obstructed	24,670 (72.15)
two or more vessel obstructed	9523 (27.85)
coronary artery bypass graft (CABG) history (yes), n (%)	188 (0.55)
percutaneous coronary intervention (PCI) history (yes), n (%)	9606 (28.09)
Physician Characteristic	
Gender (male), n (%)	32,988 (96.48)
Age, mean (S.D)	43.57 (6.81)
Physician volume, mean (S.D)	
In the previous year	116.14 (104.11)
within the study period	120.11 (103.30)
Hospital characteristic	
Geographic Location, n (%)	
Taipei	12,558 (36.73)
Northern	3652 (10.68)
Central	6514 (19.05)
Southern	5912 (17.29)
Kao-Ping	4553 (13.32)
Eastern	1004 (2.93)
Accreditation’s Status, n (%)	
Medical centers	17,456 (51.05)
Regional hospitals	15,932 (46.59)
Community hospitals	805 (2.36)
Ownership, n (%)	
Public	9305 (27.22)
Private	1889 (5.52)
Not-for-profit	22,999 (67.26)
Hospital Volume, Mean (S.D)	
In the previous year	776.08 (497.24)
Within the study period	814.40 (520.33)

**Table 3:** Patient distribution among categorization methods with different definitions of service volume.

	AHA	K-Means	GAM
**Definition 1**			
Hospital			
Low volume, n (%)	3678 (10.8)	17 (0.1)	3377 (9.9)
Medium volume, n (%)		1710 (5.0)	19,581 (57.3)
High Volume, n (%)	30,515 (89.2)	32,466 (94.0)	11,235 (32.8)
Physician			
Low volume, n (%)	9131 (26.7)	1015 (3.0)	522 (1.5)
Medium volume, n (%)		7604 (22.2)	4703 (13.8)
High Volume, n (%)	25,062 (74.3)	25,574 (74.8)	28,968 (84.7)
**Definition 2**			
Hospital			
Low volume, n (%)	3109 (9.1)	175 (0.5)	3528 (10.3)
Medium volume, n (%)		8173 (23.9)	20,402 (59.7)
High Volume, n (%)	31,084 (90.9)	25,845 (75.6)	10,263 (30.0)
Physician			
Low volume, n (%)	8504 (24.9)	926 (2.7)	496 (1.5)
Medium volume, n (%)		5272 (15.4)	3342 (9.8)
High Volume, n (%)	25,689 (75.1)	27,995 (81.9)	30,355 (88.8)

Definition 1: the cumulative service volumes by each physician and hospital in a previous year for each treatment. Definition 2: the cumulative operation volumes by each physician and hospital within the study period.

**Table 4:** Results of the multilevel analysis with hospital service volume.

	Model 1 (AHA 2013)			Model 2 (K-Means)			Model 3 (GAM)		
	OR	95% C.I.		OR	95% C.I.		OR	95% C.I.	
		LCL	UCL		LCL	UCL		LCL	UCL
**Definition 1**									
Hospital volume									
Low	1.01	0.75	1.35	1.01	0.69	1.49	1.21	0.90	1.63
AIC	7711.09			7711.20			7709.57		
**Definition 2**									
Hospital volume									
Low	1.25	0.90	1.75	1.21	0.94	1.56	1.23	0.89	1.70
AIC	7709.51			7708.80			7709.59		

Definition 1: the cumulative service volumes by each physician and hospital in a previous year for each treatment. Definition 2: the cumulative operation volumes by each physician and hospital within the study period. OR: odds ratio; C.I.: confidence interval; LCL: lower confidence limit; UCL: upper confidence limit; AIC: Akaike information criterion. All models were adjusted by patient’s age, Comorbidity Index, stent use, prior PCI treatment, physician’s age, and hospital ownership, location, and accreditation status.

**Table 5:** Results of the multilevel analysis with hospital and physician service volume.

	Model 1 (AHA)			Model 2 (K-Means)			Model 3 (GAM)		
	OR	95% C.I.		OR	95% C.I.		OR	95% C.I.	
		LCL	UCL		LCL	UCL		LCL	UCL
**Definition 1**									
Hospital volume									
Low	0.91	0.70	1.19	0.87	0.61	1.24	1.11	0.86	1.42
Physician volume									
Low	2.34	1.92	2.84	2.37	1.95	2.87	2.75	2.27	3.31
AIC	7648.69			7645.18			7614.32		
**Definition 2**									
Hospital volume									
Low	1.03	0.77	1.37	1.16	0.94	1.43	1.13	0.87	1.47
Physician volume									
Low	2.73	2.223	3.343	2.78	2.29	3.39	3.15	2.59	3.83
AIC	7634.17			7624.48			7602.20		

Definition 1: the cumulative service volumes by each physician and hospital in a previous year for each treatment. Definition 2: the cumulative operation volumes by each physician and hospital within the study period; OR: odds ratio; C.I.: confidence interval; LCL: lower confidence limit; UCL: upper confidence limit. All models were adjusted by patient’s age, Comorbidity Index, stent use, prior PCI treatment, physician’s age, and hospital ownership, location, and accreditation status.

**Table 6:** Results of stratified analysis: AHA.

	High Hospital Volume			Low Hospital Volume		
	Model 1 (AHA 2013)			Model 1 (AHA 2013)		
	OR	95% C.I.		OR	95% C.I.	
		LCL	UCL		LCL	UCL
**Definition 1**						
Physician volume						
Low	2.32	1.87	2.88	2.30	1.43	3.69
AIC	6553.17			1112.53		
**Definition 2**						
Physician volume						
Low	2.77	2.22	3.45	2.15	1.30	3.56
AIC	6620.08			1027.72		

Definition 1: the cumulative service volumes by each physician and hospital in a previous year for each treatment. Definition 2: the cumulative operation volumes by each physician and hospital within the study period. OR: odds ratio; C.I.: confidence interval; LCL: lower confidence limit; UCL: upper confidence limit. All models were adjusted by patient’s age, Comorbidity Index, stent use, prior PCI treatment, physician’s age, and hospital ownership, location, and accreditation status.

**Table 7:** Results of stratified analysis: k-means.

	High Hospital Volume			Low Hospital Volume		
	Model 2 (K-Means)			Model 2 (K-Means)		
	OR	95% C.I.		OR	95% C.I.	
		LCL	UCL		LCL	UCL
**Definition 1**						
Physician volume						
Low	2.40	1.96	2.94	1.65	0.82	3.34
AIC	7094.94			564.84		
**Definition 2**						
Physician volume						
Low	2.72	2.11	3.49	2.85	2.07	3.93
AIC	5237.73			2400.92		

Definition 1: the cumulative service volumes by each physician and hospital in a previous year for each treatment. Definition 2: the cumulative operation volumes by each physician and hospital within the study period. OR: odds ratio; C.I.: confidence interval; LCL: lower confidence limit; UCL: upper confidence limit. All models were adjusted by patient’s age, Comorbidity Index, stent use, prior PCI treatment, physician’s age, and hospital ownership, location, and accreditation status.

**Table 8:** Results of stratified analysis: GAM.

	High Hospital Volume			Low Hospital Volume		
	Model 3 (GAM)			Model 3 (GAM)		
	OR	95% C.I.		OR	95% C.I.	
		LCL	UCL		LCL	UCL
**Definition 1**						
Physician volume						
Low	3.41	2.09	5.56	2.60	2.12	3.19
AIC	1750.48			5860.39		
**Definition 2**						
Physician volume						
Low	4.93	2.80	8.70	2.90	2.36	3.56
AIC	1480.01			6113.56		

Definition 1: the cumulative service volumes by each physician and hospital in a previous year for each treatment. Definition 2: the cumulative operation volumes by each physician and hospital within the study period; OR: odds ratio; C.I.: confidence interval; LCL: lower confidence limit; UCL: upper confidence limit. All models were adjusted by patient’s age, Comorbidity Index, stent use, prior PCI treatment, physician’s age, and hospital ownership, location, and accreditation status.
